# Oncological outcomes of Crohn's disease‐associated cancers focusing on disease behavior

**DOI:** 10.1002/ags3.12653

**Published:** 2023-01-18

**Authors:** Akira Yamamoto, Yuji Toiyama, Hiroki Ikeuchi, Motoi Uchino, Kitaro Futami, Kinya Okamoto, Takayuki Ogino, Soichiro Ishihara, Yoichi Ajioka, Kenichi Sugihara, for the Study Group for Inflammatory Bowel Disease Associated Intestinal Cancers by the Japanese Society for Cancer of the Colon and Rectum

**Affiliations:** ^1^ Department of Gastrointestinal and Pediatric Surgery, Division of Reparative Medicine Institute of Life Sciences, Mie University Graduate School of Medicine Tsu Japan; ^2^ Department of Inflammatory Bowel Disease Surgery Hyogo College of Medicine Nishinomiya Japan; ^3^ Department of Surgery Fukuoka University Chikushi Hospital Chikushino Japan; ^4^ Department of Coloproctology Tokyo Yamate Medical Center Tokyo Japan; ^5^ Department of Gastroenterological Surgery, Graduate School of Medical Osaka University Osaka Japan; ^6^ Department of Surgical Oncology The University of Tokyo Tokyo Japan; ^7^ Division of Molecular and Diagnostic Pathology, Graduate School of Medical and Dental Sciences Niigata University Niigata Japan; ^8^ Tokyo Medical and Dental University Tokyo Japan

**Keywords:** Crohn's disease, Crohn's disease‐associated cancer, disease behavior, penetrating, stricturing

## Abstract

**Background:**

The overall risk of colorectal cancer in Crohn's disease (CD) is higher than in the general population, and CD‐associated cancer (CDAC) has poorer prognosis than sporadic cancer. Developing treatment strategies for improving the prognosis of CDAC, we evaluated the characteristics of CDAC according to the underlying disease behavior, namely stricturing and penetrating.

**Methods:**

This multicenter retrospective study comprises 316 CDAC patients who underwent surgery between 1985 and 2019. Clinicopathological findings including disease behavior and oncological outcomes were investigated.

**Results:**

There was no association between the preoperative course of CDAC patients and disease behavior; however, postoperative information revealed distinctly different characteristics between CDAC patients with stricturing behavior and those with penetrating behavior (stricturing with lymphatic invasion and peritoneal dissemination recurrence, and penetrating with histologically poorly differentiated and local recurrence). Oncological outcome of patients with CDAC was distinctly different according to disease behavior, as penetrating provided a poor outcome (overall survival [OS]: *p* = 0.02; relapse‐free survival [RFS]: *p* = 0.002) whereas stricturing had no effect. Furthermore, penetrating behavior was identified as one of the independent risk factors for poor OS and RFS (OS: hazard ratio [HR] 1.89, 95% confidence interval [CI] 1.16–3.09, *p* = 0.01; RFS: HR 2.15, 95% CI 1.28–3.63, *p* = 0.004).

**Conclusions:**

Our study highlights the different characteristics of CDAC according to the underlying disease behavior and substantiates the poor prognosis of CDAC patients with penetrating behavior. Treatment planning including screening, surgical procedures, and postoperative treatment, with awareness of these findings, may contribute to improved prognosis for CDAC patients.

## Graphical abstract

Time‐to‐event analysis showed that disease behavior affected the oncological outcome of CDAC patients. Although stricturing behavior was not associated with OS and DFS, penetrating behavior was identified as bringing about a poor oncological outcome for both OS and DFS.

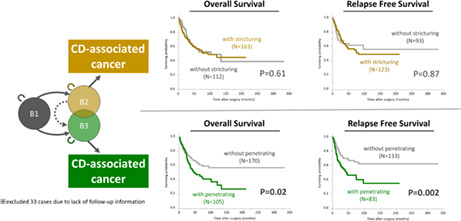

## 1 INTRODUCTION

One of the many problems surrounding the research field of inflammatory bowel disease is development of cancer against the background of chronic persistent inflammation. Accumulating evidence has shown the increased risk of cancer for patients with Crohn's disease (CD) who have had extensive, long‐standing, unresected involvement.^1^, ^2^, ^3^, ^4^ CD‐associated cancer (CDAC) can develop in diverse sites such as small bowel, colon, rectum, anal canal, and anal fistula, and the prognosis of CDAC is poorer than that of sporadic cancer.^5^, ^6^ In terms of exposure to chronic persistent inflammation, recent therapeutic advances represented by biologics decrease the risk of surgery and indicate a potential to increase the number of patients with long‐standing CD, implying a possible increase in those with CDAC.

Disease behavior such as stenosis and fistula, which is one of the features of CD, is considered to be one of the phenotypes of chronic persistent inflammation in the local environment. According to the Montreal classification, CD consists of three components: age at diagnosis, location, and behavior.^7^ Changes in lesion location (L1, ileal; L2, colonic; L3, ileocolonic; L4, isolated upper disease) during long‐term disease are rare; however, changes in disease behavior (B1, nonstricturing, nonpenetrating; B2, stricturing; B3, penetrating) are often seen over the long‐term disease course.^8^ Indeed, B1 cases at the time of diagnosis can develop high rates of B2 or B3 disease behavior during the period of disease.^9^

The factors responsible for either B2 or B3 complications as a result of chronic persistent inflammation remain unclear, although B2 is thought to occur through fibrosis when regeneration and repair cannot restore normal structure.^10^, ^11^ By contrast, B3 is thought to occur directly as a result of active inflammation of the intestinal wall.^12^ The causes of both behaviors assume a common process of chronic inflammation, and the phenomenon of migration from B2 to B3 can occur; however, these are classified as different pathological conditions, and the differential course of CD can affect the differential pathology.^13^

Considering that disease behavior can represent phenotypes of chronic persistent inflammation, B2 or B3 lesions may affect carcinogenesis, known as the dysplasia‐carcinoma sequence. Therefore, we hypothesized that different disease behaviors can provide differing clinical phenotypes of CDAC, which has carcinogenic predisposition against the background of chronic inflammation. In this study, we evaluated the characteristics of CDAC according to the underlying disease behavior and examined the possibility of clinical applications for improving the prognosis of CDAC.

## 2 MATERIALS AND METHODS

### 2.1 Patients

**FIGURE 1:**
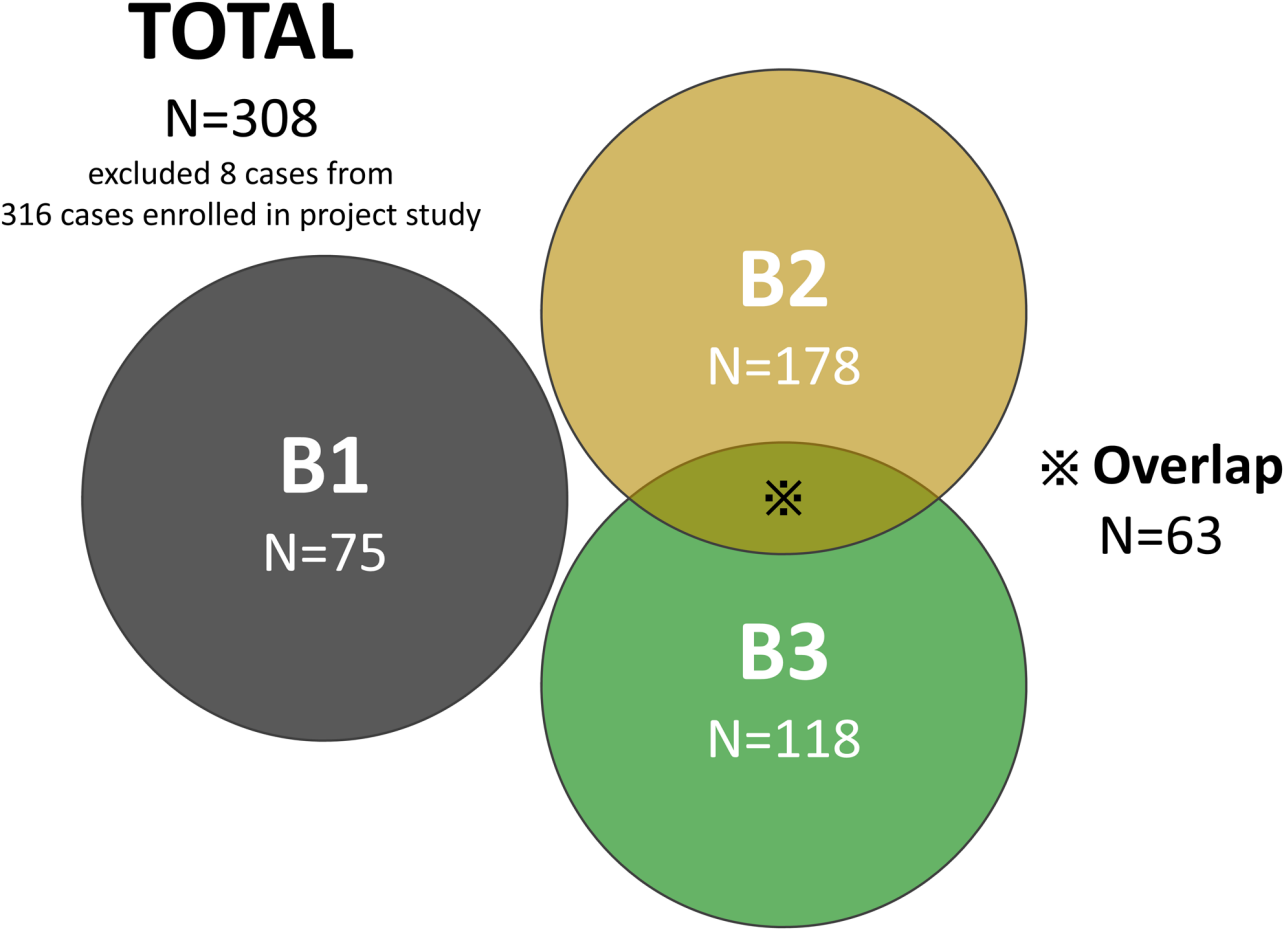
A total of 316 CDAC patients who underwent surgery between 1985 and 2019 were enrolled in the project study. From the data collected, 308 cases, excluding those without information on disease behavior, were examined.

A total of 316 CDAC patients who underwent surgery between 1985 and 2019 were enrolled in the project study led by the Japanese Society for Cancer of Colon and Rectum, which collected the data from 39 participating institutions.^14^ This multicenter retrospective study was approved with the permission of The University of Tokyo Ethics Review Board (2019220NI‐(2)). From the data collected, after excluding those without information on disease behavior, 308 cases were examined (Figure 1). We collected information about patients' baseline characteristics, diagnostic procedures, treatment details, histopathological findings, and long‐term oncological outcomes.

### 2.2 Definition and interpretation of terms

In this study, the disease behavior was basically classified at the time of surgery that led to cancer diagnosis. Stricturing was defined as fibrostenotic features and penetrating as intra‐abdominal or perianal fistula, perianal ulcer, inflammatory mass, and/or abscess. Although these behaviors were mainly determined from intra‐operative findings, but also comprehensively from preoperative diagnostic studies such as small bowel series, enterographic contrast, as well as magnetic resonance imaging or from postoperative pathological evidence.^15^ Histopathological diagnosis was confirmed for enrolled patients based on surgical specimens, including histological differentiation, T stage, venous invasion, lymphatic invasion, node involvements. These were defined based on TNM classification and Japanese Classification of Colorectal, Appendiceal, and Anal Carcinoma.^16^ In this study, histological differentiation were classified into two groups, well to moderately differentiated and others. The “well to moderately differentiated type” includes well differentiated type (tub1) and moderately differentiated type (tub2), on the other hand, “others” includes histological findings of mucinous adenocarcinoma (muc, 67.8%), signet‐ring cell carcinoma (sig, 10.1%), poorly differentiated adenocarcinoma (por, 8.9%), and the other histology types (13.2%). Recurrence of cancer was diagnosed by imaging evaluation such as endoscopy, computed tomography (CT), magnetic resonance imaging (MRI), and 18F‐fluorodeoxyglucose positron emission tomography (FDG‐PET/CT) at the postoperative surveillance period. Masses along major vessels were diagnosed to be mainly lymph node recurrences based on their tendency to enlarge and/or FDG accumulation, while masses on residual intestinal tract such as anastomosis and dissected surfaces such as the anterior sacral surface and lateral pelvic wall were diagnosed to be local recurrences based on their tendency to enlarge and/or FDG accumulation. Other recurrences such as peritoneal dissemination recurrence, liver metastasis, and lung metastasis were similarly diagnosed by imaging findings.

### 2.3 Statistical analysis

Comparisons of patients' and clinical characteristics between groups were evaluated using the Kruskal–Wallis test for continuous variables (expressed as median ± interquartile range) and Pearson's chi‐squared test or Fisher's exact test for categorical variables, as appropriate. A survival analysis was performed using the Kaplan–Meier method and was compared using the log‐rank test. Overall survival (OS) was measured from the date the patient underwent surgery until the date of death resulting from any cause, or until the last known follow‐up in patients who were still alive. Relapse‐free survival (RFS) was measured from the date the patient underwent surgery to the date of disease recurrence, death from any cause (i.e., cancer‐unrelated deaths were not censored), or until last contact with the patient. In survival analysis, we excluded 33 patients because of a lack of follow‐up information. Cox's proportional hazards model was used to estimate hazard ratio (HR) for OS and RFS. Clinical variables that were considered for univariate and multivariate analyses, in addition to disease behavior, were previously identified confounding factors with an impact on clinical course of CD: onset age, disease duration, smoking history, ileum lesion, colon lesion, use of 5‐aminosalicylic acid, use of steroids, and use of anti‐tumor necrosis factor‐α drugs; and factors with an impact on prognosis in patients with colorectal cancer: sex, age at diagnosis of cancer, histological differentiation (well to moderately differentiated or others), pathological T stage (T1/2 or T3/4), lymphatic invasion (present or absent), venous invasion (present or absent), and lymph node metastasis (present or absent). All *p* values were two‐sided, and *p* < 0.05 was considered statistically significant. Statistical analyses were performed using JMP Pro 13.1 (SAS Institute).

## 3 RESULTS

### 3.1 Patients' characteristics before diagnosis of cancer

Patients' characteristics and clinical courses before diagnosis of cancer are shown in Table 1. There was no association between the clinical course of CDAC patients and the presence of stricturing behavior. Similarly, there was no association between clinical course and the presence of penetrating behavior. However, it has the significant relationship between location of cancer and disease behavior, and between type of surgery and disease behavior, respectively. Reflecting the large number of cases with lesions in anus and anal canal, abdominoperineal resection was often selected as the operative procedure.

**TABLE 1:** Association between disease behavior and clinical course before diagnosis of cancer

	Stricturing		*p* value	Penetrating		*p* value
	Absence	Presence		Absence	Presence	
Clinical factor						
Sex						
Male	86 (66.2)	120 (67.4)	0.82	125 (65.8)	81 (68.6)	0.60
Female	44 (33.8)	58 (32.6)		65 (34.2)	37 (31.4)	
Age at onset of CD						
≥25 (median)	68 (53.1)	85 (48.0)	0.38	101 (53.7)	52 (44.4)	0.12
<25	60 (46.9)	92 (52.0)		87 (46.3)	65 (55.6)	
Disease duration (months)						
≥240 (median)	59 (48.4)	83 (51.9)	0.56	83 (48.0)	59 (54.1)	0.31
<240	63 (54.6)	77 (48.1)		90 (52.0)	50 (48.9)	
Age at cancer diagnosis						
≥46 (median)	67 (52.8)	87 (51.2)	0.79	97 (53.3)	57 (49.6)	0.53
<46	60 (47.2)	83 (48.8)		85 (46.7)	58 (50.4)	
Smoking history						
No	74 (71.2)	112 (77.2)	0.28	107 (70.4)	79 (81.4)	0.05
Yes	30 (28.8)	33 (22.8)		45 (29.6)	18 (18.6)	
Receive 5‐ASA therapy within 1 year before surgery						
No	23 (21.7)	40 (28.8)	0.21	45 (29.4)	18 (19.6)	0.09
Yes	83 (78.3)	99 (71.2)		108 (70.6)	74 (80.4)	
Receive systemic steroid within 1 year before surgery						
No	78 (78.8)	101 (80.2)	0.80	111 (79.9)	68 (79.1)	0.89
Yes	21 (21.2)	25 (19.8)		28 (20.1)	18 (20.9	
Receive anti‐TNF within 1 year before surgery						
No	4 (11.8)	11 (21.2)	0.26	9 (16.7)	6 (18.8)	0.81
Yes	30 (88.2)	41 (78.9)		45 (83.3)	26 (81.2)	
History of surgery for CD						
No	43 (39.8)	59 (41.8)	0.75	71 (45.2)	31 (33.7)	0.07
Yes	65 (60.2)	82 (58.2)		86 (54.8)	61 (66.3)	
Location of cancer						
Small intestine	7 (6.0)	25 (15.2)	0.03*	19 (10.7)	13 (12.8)	<0.0001*
Colon	19 (16.5)	24 (14.6)		37 (20.8)	6 (5.9)	
Rectum	30 (25.9)	52 (31.7)		61 (34.3)	21 (20.6)	
Anal canal/anus	60 (51.7)	63 (38.4)		61 (34.3)	62 (60.8)	
Type of surgery						
Small intestinal resection/ileocecal resection	4 (3.3)	17 (10.0)	0.02*	18 (10.0)	3 (2.7)	0.0003*
Colectomy	23 (18.9)	33 (19.4)		45 (25.0)	11 (9.8)	
Anterior resection	19 (14.6)	18 (10.6)		23 (12.8)	14 (12.5)	
Abdominoperineal resection	59 (48.3)	61 (35.9)		64 (35.6)	56 (50.0)	
Others	17 (13.9)	41 (24.1)		30 (16.7)	28 (25.0)	

*Note*: * and bold denote statistical significance with *p* value < 0.05.

Abbreviations: 5‐ASA, 5‐aminosalicylic acid; CD, Crohn's disease; TNF, tumor necrosis factor.

### 3.2 Difference in Postoperative Information for CDAC Patients According to CD Behavior

Postoperative information, which included pathological findings and recurrence type, are shown in Table 2. Contrary to preoperative information, the postoperative information suggested that each behavior had a different impact on pathological factors and the postoperative course of patients with CDAC. Stricturing behavior was associated with high T stage (*p* = 0.002), presence of lymphatic invasion (*p* = 0.03), and high TNM stage (*p* = 0.002). Penetrating behavior was associated with poorly differentiated cancer (*p* = 0.02) and high T stage (*p* = 0.03). Interestingly, the recurrence type in patients with CD‐associated cancer was shown to be different for each behavior. Specifically, stricturing behavior was associated with recurrence of peritoneal dissemination (*p* = 0.04), whereas penetrating behavior was associated with local recurrence (*p* = 0.008).

**TABLE 2:** Association between disease behavior and pathological factors or recurrence type

	Stricturing		*p* value	Penetrating		*p* value
	Absence	Presence		Absence	Presence	
Pathological factor						
Histology						
Well to mod	55 (50.5)	67 (43.2)	0.25	84 (52.2)	38 (36.9)	0.02*
Others	54 (49.5)	88 (56.8)		77 (47.8)	65 (63.1)	
T stage						
T0/1/2	45 (40.2)	34 (23.0)	0.003*	57 (35.2)	22 (22.5)	0.03*
T3/4	67 (59.8)	114 (77.0)		105 (64.8)	76 (77.5)	
Venous invasion						
Presence	68 (61.8)	77 (55.4)	0.31	91 (58.0)	54 (58.7)	0.91
Absence	42 (38.4)	62 (44.6)		66 (42.0)	38 (41.3)	
Lymphatic invasion						
Presence	77 (49.4)	33 (35.5)	0.03*	94 (59.9)	62 (67.4)	0.23
Absence	79 (50.6)	60 (64.5)		63 (40.1)	30 (32.6)	
Node involvement						
Presence	77 (71.3)	97 (68.8)	0.67	106 (66.7)	68 (75.6)	0.14
Absence	31 (28.7)	44 (31.2)		53 (33.3)	22 (24.4)	
TNM Stage						
0/I	41 (36.3)	27 (19.3)	0.002*	50 (30.3)	18 (20.5)	0.09
II/III/IV	72 (63.7)	113 (80.7)		115 (69.7)	70 (79.5)	
Recurrence type						
Local						
Absence	19 (50.0)	30 (51.7)	0.87	31 (64.6)	18 (37.5)	0.008*
Presence	19 (50.0)	28 (48.3)		17 (35.4)	30 (62.5)	
Peritoneum						
Absence	34 (91.9)	46 (73.0)	0.04*	39 (75.0)	41 (85.4)	0.22
Presence	3 (8.1)	17 (27.0)		13 (25.0)	7 (14.6)	
Lymph node						
Absence	31 (83.8)	53 (89.8)	0.53	40 (81.6)	44 (93.6)	0.12
Presence	6 (16.2)	6 (10.2)		9 (18.4)	3 (6.4)	
Liver						
Absence	34 (91.9)	53 (91.4)	1.00	43 (89.6)	44 (93.6)	0.71
Presence	3 (8.1)	5 (8.6)		5 (10.4)	3 (6.4)	
Lung						
Absence	32 (86.5)	47 (81.0)	0.58	39 (79.6)	40 (87.0)	0.42
Presence	5 (13.5)	11 (19.0)		10 (20.4)	6 (13.0)	

*Note*: * and bold denote statistical significance with *p* value < 0.05.

Abbreviations: mod, moderately differentiated; TNM, tumor node metastasis; well, well differentiated.

### 3.3 Oncological outcome of CDAC patients according to CD behavior

**FIGURE 2:**
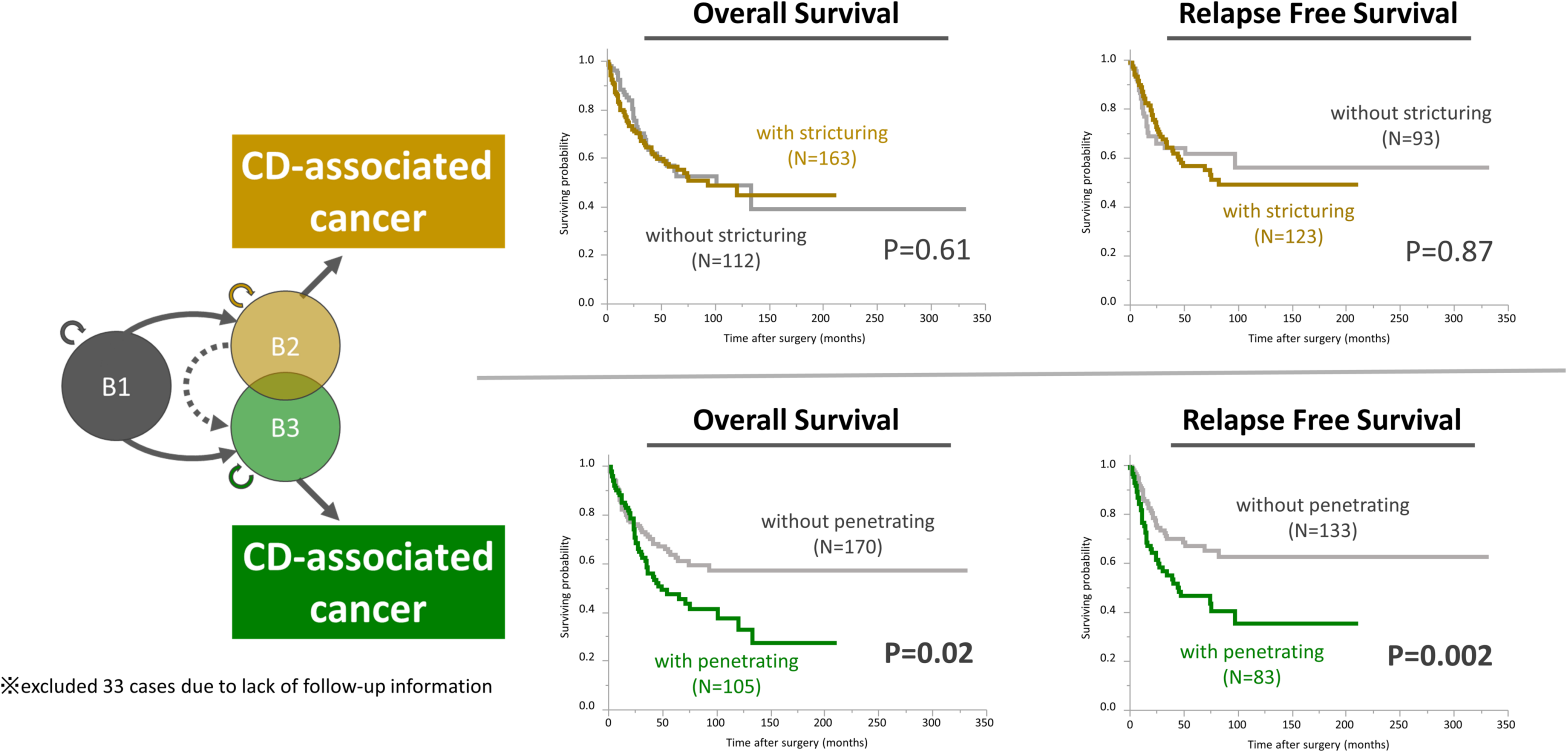
CD behavior affects the oncological outcome of CDAC patients. Whereas stricturing behavior was not associated with overall survival (OS) and relapse‐free survival (RFS), penetrating behavior was identified as bringing about a poor oncological outcome for both OS and RFS.

We next investigated the impact of CD behavior on the oncological outcomes. Time‐to‐event analysis showed that disease behavior affected the oncological outcome of CDAC patients (Figure 2). Although stricturing behavior was not associated with OS and RFS, penetrating behavior was identified as bringing about a poor oncological outcome for both OS and RFS (OS, *p* = 0.02; RFS, *p* = 0.002; log‐rank test).

### 3.4 Identified factors associated with poor prognosis for CDAC patients

We further evaluated the predictive factors for oncological outcomes of CDAC. Variables to be evaluated comprised CD characteristics and the clinical course, including disease behavior and well‐known risk factors for poor oncological outcome. Notably, along with cancer histology of poor differentiation, high T stage, venous invasion, and node involvement, penetrating behavior was identified as an independent risk factor for poor OS (HR 1.89, 95% confidence interval [CI] 1.16–3.09, *p* = 0.01; Table 3). In addition, penetrating behavior was identified as an independent risk factor for poor RFS (HR 2.15, 95% CI 1.28–3.63, *p* = 0.004; Table 3) along with high T stage and node involvement.

**TABLE 3:** Univariate and multivariate analysis of predictors for oncological outcome

	Univariate analysis			Multivariate analysis		
	HR	95% CI	*p* value	HR	95% CI	*p* value
For overall survival						
Sex						
Male	0.96	0.65–1.43	0.84			
Age at onset of CD						
≥Median	0.77	0.52–1.12	0.17			
Disease duration						
≥Median	1.24	0.83–1.86	0.31			
Age at diagnosis of Cancer						
≥Median	0.70	0.48–1.03	0.07			
Smoking history						
Yes	1.47	0.90–2.33	0.12			
Ileum lesion						
Presence	1.36	0.83–2.37	0.23			
Colon lesion						
Presence	0.91	0.57–1.54	0.71			
Stricturing						
Presence	1.11	0.75–1.65	0.61			
Penetrating						
Presence	1.56	1.06–2.30	0.02*	1.89	1.16–3.09	0.01*
Use of 5‐ASA						
Yes	0.73	0.45–1.20	0.20			
Use of Steroid						
Yes	0.93	0.52–1.56	0.78			
Use of anti‐TNF						
Yes	1.01	0.34–4.31	0.99			
Histology						
Others	2.76	1.81–4.33	<0.0001*	1.92	1.16–3.28	0.01*
T factor						
T3/4	6.42	3.18–15.3	<0.0001*	3.03	1.42–7.49	0.003*
Lymphatic invasion						
Presence	3.01	1.91–4.83	<0.0001*	1.04	0.53–2.03	0.91
Venous invasion						
Presence	3.36	2.14–5.34	<0.0001*	2.01	1.02–3.95	0.04*
Node involvement						
Presence	3.21	2.09–4.92	<0.0001*	1.92	1.11–3.31	0.02*
For relapse‐free survival						
Sex						
Male	0.90	0.58–1.42	0.64			
Age at onset of CD						
≥Median	0.79	0.51–1.22	0.28			
Disease duration						
≥Median	0.81	0.50–1.30	0.39			
Age at diagnosis of Cancer						
≥Median	0.57	0.37–0.90	0.01*	0.95	0.53–1.71	0.87
Smoking history						
Yes	0.85	0.45–1.50	0.59			
Ileum lesion						
Presence	1.19	0.71–2.12	0.53			
Colon lesion						
Presence	0.72	0.42–1.34	0.28			
Stricturing						
Presence	1.04	0.66–1.65	0.87			
Penetrating						
Presence	1.96	1.26–3.07	0.003*	2.15	1.28–3.63	0.004*
Use of 5‐ASA						
Yes	0.71	0.41–1.31	0.26			
Use of Steroid						
Yes	1.26	0.68–2.22	0.45			
Use of anti‐TNF						
Yes	1.73	0.50–10.9	0.43			
Histology						
Others	2.60	1.62–4.33	<0.0001*	1.60	0.88–2.98	0.13
T factor						
T3/4	5.88	3.00–13.3	<0.0001*	3.64	1.71–8.98	0.0004*
Lymphatic invasion						
Presence	2.60	1.63–4.17	<0.0001*	1.38	0.71–2.69	0.34
Venous invasion						
Presence	2.54	1.60–4.06	0.0001*	0.98	0.50–1.93	0.96
Node involvement						
Presence	3.70	2.34–5.87	<0.0001*	2.21	1.23–4.00	0.008*

*Note*: * and bold denote statistical significance with *p* value < 0.05.

Abbreviations: 5‐ASA, 5‐aminosalicylic acid; CD, Crohn's disease; TNF, tumor necrosis factor.

### 3.5 Assessment of disease behaviors with both stricture and penetration

**FIGURE 3:**
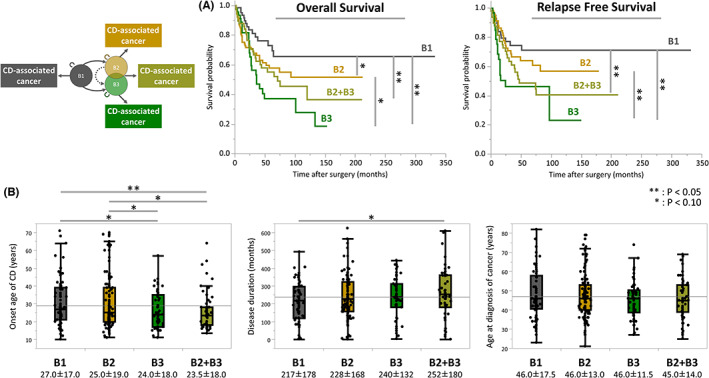
(A) We also examined this cohort subdivided without duplication, categorized as: B1, nonstricturing and nonpenetrating; B2, stricturing; B3, penetrating; and B2 + B3, stricturing and penetrating. CDAC patients with B3 and B2 + B3 had significantly poor OS compared with those with B1. Moreover, those with B3 had significantly poor RFS compared with B1 and B2, while those with B2 + B3 had poor RFS compared with B1. (B) the association between age‐related factors and behaviors showed that onset age of CD in B2 + B3‐CDAC was significantly younger than in B1‐CDAC, and disease duration of CD in B2 + B3‐CDAC tended to be longer than that in B1‐CDAC.

The previous evaluations in this study were based on the presence or absence of stricturing or penetrating behavior. We next examined this cohort subdivided without duplication, categorized according to the Paris criteria: B1, nonstricturing and nonpenetrating; B2, stricturing; B3, penetrating; and B2 + B3, stricturing and penetrating.^17^ Time‐to‐event analysis showed that CDAC patients with B3 and B2 + B3 had significantly poor OS compared with those with B1 (B3‐CDAC vs. B1‐CDAC: *p* = 0.001; B2 + B3‐CDAC vs. B1‐CDAC: *p* = 0.05; Figure 3). Moreover, those with B3 had significantly poor RFS compared with B1 and B2 (B3‐CDAC vs. B1‐CDAC: *p* = 0.003; B3‐CDAC vs. B2‐CDAC: *p* = 0.01), and those with B2 + B3 also had poor RFS compared with those with B1 (B2 + B3‐CDAC vs. B1‐CDAC: *p* = 0.03; Figure 3). It is noteworthy that the Kaplan–Meier curve of B2 + B3‐CDAC was located almost between that of B2‐CDAC and B3‐CDAC. Furthermore, the association between age‐related factors and behaviors showed that onset age of CD in B2 + B3‐CDAC was significantly younger than for B1‐CDAC, and disease duration of CD in B2 + B3‐CDAC tended to be longer than that of B1‐CDAC (Figure 3). Notably, in terms of medians, CDAC patients with B2 + B3 but not B3 had the youngest age at onset of CD, longest disease duration, and youngest age at diagnosis of cancer.

## 4 DISCUSSION

Recent therapeutic advances indicate a potential to increase the number of patients with long‐standing CD, which has carcinogenic predisposition against the background of chronic inflammation, implying a possible concomitant increase in those with CDAC. The overall combined risk of colorectal cancer in CD is more than two and a half times that of the general population, and the cumulative risk of colorectal cancer 10 years after diagnosis of CD at any site is 2.9%, as previously reported.^1^ Provision of a treatment strategy for CDAC is an urgent issue, and it is desirable to develop tools that will be useful in decision‐making about treatment strategies such as screening for early detection of cancer, selection of surgical procedures with a future vision of the expected postoperative course, and postoperative therapy. However, the biggest challenge in the development of such a tool is the rarity of CDAC. Since there are only a few reports of CDAC, it is meaningful to evaluate this rare condition by collecting a substantial number of cases as we have done in this study.

We evaluated the characteristics of CDAC according to the underlying disease behavior, using the hypothesis that different disease behaviors can provide a differing clinical phenotype of CDAC. During the course of this study, several novel findings were demonstrated regarding the association between clinicopathological findings of CDAC and underlying disease behavior of CD. First, we showed that pathological findings and recurrence type revealed distinctly different characteristics between CDAC patients with stricturing behavior and those with penetrating behavior. Second, the oncological outcome of patients with CDAC was distinctly different according to the disease behavior, whereby penetrating behavior led to a poor outcome whereas stricturing did not affect the outcome. Third, penetrating behavior was identified as one of the independent risk factors for poor OS and RFS, whereas stricturing was not a risk factor. Finally, CD with both stricturing and penetrating behavior was suggested to be an independent disease phenotype.

The main result of our study is that different disease behaviors can provide different clinical phenotypes of CDAC. We speculate that differences in the aspect of inflammation led to differences in the carcinogenic process. It is possible that the difference in inflammation that leads to fibrosis in B2^10^, ^11^ and direct damage to the intestinal epithelium in B3^12^ may have brought about different characteristics of cancer, the cancer generated from B2‐CD mucosa being lymphatic invasive and that from B3‐CD mucosa being poorly differentiated. Although fibrotic lesions increase the risk of cancer in several tissues,^18^, ^19^ the mechanism linking fibrosis and cancer is unclear. Accumulating evidence indicates that YAP/TAZ signaling can modulate the fibrotic response as well as the behavior of cancer cells, which maintains crosstalk with transforming growth factor β and Wnt signaling pathways associated with both fibrosis and cancer.^20^, ^21^, ^22^ This evidence is not applicable to gastrointestinal cancer, although similar mechanisms may be involved in the development of CDAC in B2‐CD mucosa. However, cancer of the anal fistula, a form of CDAC in B3‐CD mucosa, is thought to be caused by the straying of epithelial components from the perianal crypt into the fistula by an unidentified mechanism, leading to carcinogenesis through adenomatous hyperplastic changes associated with chronic inflammation.^23^ A similar phenomenon occurring outside the anal cavity may also lead to the development of CDAC with a poor prognosis.

A further result of our study is that the behavior of CD responsible for cancer affects the oncological outcome. Differences in the characteristics of cancer may result in a difference in the recurrence type and the oncological outcome. Multivariate analysis indeed clarified that not stricturing but penetrating behavior is one of the independent risk factors for OS and RFS. The fact that this was extracted alongside the well‐known risk factors shows the impact of this concept. However, there were also cases through surgery as diagnosis of benign lesion in which malignancy was identified incidentally in the postoperative pathological findings (Table 4), and it is one of the limitations of this study that surgery is not always preceded by the assumption of the presence of cancer. Nevertheless, stricturing‐CDAC patients were often not operated on with malignancy in mind, and the results suggest high malignancy of penetrating CDAC, which had a poor prognosis even though it was operated on with presumed malignancy. These results were consistent with our hypothesis that differential disease behavior of CD can affect differing pathological conditions and bring about distinct phenotypes of these cancers.

**TABLE 4:** Association between disease behavior and surgical factors.

	Stricturing		*p* value	Penetrating		*p* value
	Absence	Presence		Absence	Presence	
Surgical factor						
Indication (resected as)						
Benign	23 (19.2)	70 (41.9)	<0.0001*	56 (31.5)	37 (33.9)	0.66
Malignancy	97 (80.8)	97 (58.1)		122 (68.5)	72 (66.1)	
Curativity						
R0	91 (83.5)	115 (68.9)	0.006*	130 (76.5)	76 (71.7)	0.38
R1/2	18 (16.5)	52 (31.1)		40 (23.5)	30 (28.3)	

*Note*: * and bold denote statistical significance with *p* value < 0.05.

**FIGURE 4:**
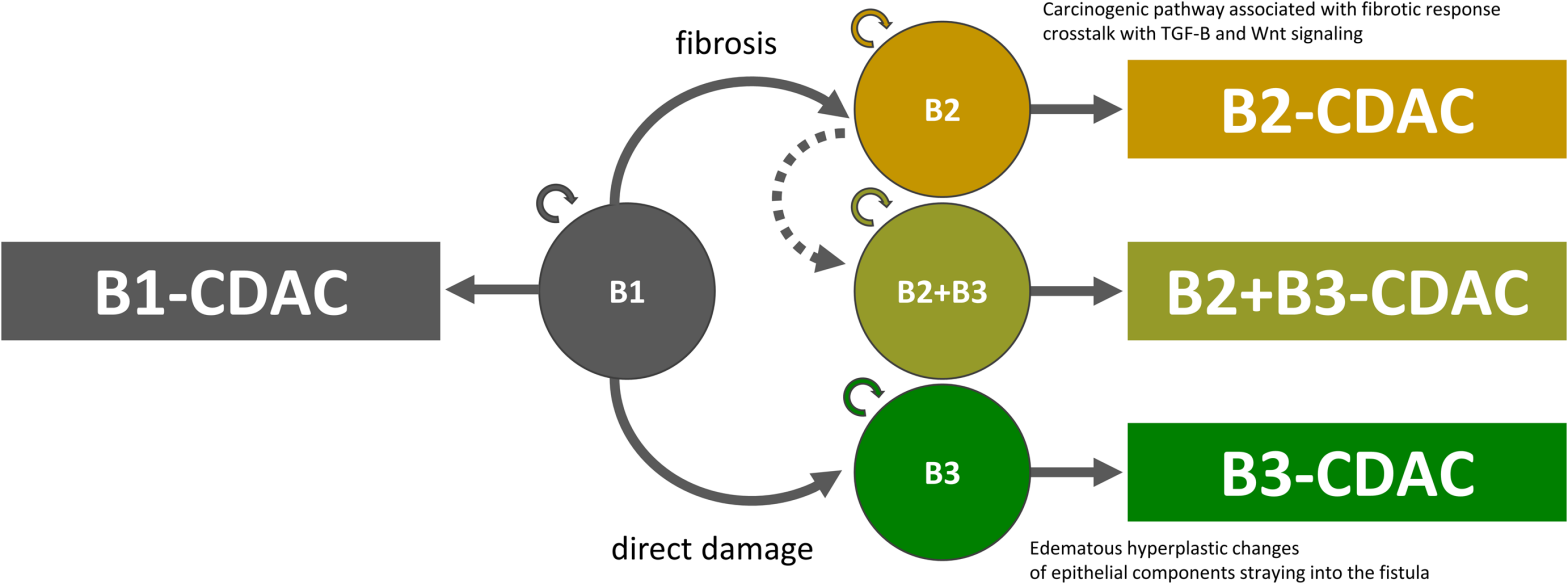
The tendency of B1, B2, B2 + B3, and B3 toward poor prognosis in that order suggests that the B2 + B3 phenotype is a migration stage from B2 to B3, but in fact B2 + B3 cases represent the group with the longest disease duration, suggesting that they may be independent disease phenotypes.

Practical classification of CDAC seemed to conflict with the conventional Montreal classification because this classification is mainly for CD as a benign disease and does not assume malignancy. However, this classification is considered to be useful for understanding the pathogenesis of CD and examination of our hypothesis. When the classification was not based on the presence or absence of stricturing and penetrating behavior but on the nonoverlapping classification of B1, B2, B3, and B2 + B3 as in the Paris criteria,^17^ each of the disease types showed time‐dependent characteristics. The tendency of B1, B2, B2 + B3, and B3 in that order toward poor prognosis suggests that the B2 + B3 phenotype is a migration stage from B2 to B3, but in fact B2 + B3 cases are those with the longest disease duration, suggesting that they may be independent disease phenotypes (Figure 4). Although this study focused on disease behavior, location of cancer is also an important factor (Tables S1 and S2). When the outcome of cases with colorectal CDAC is evaluated by disease behavior, both stricturing and penetrating cases have a poor outcome (Figure S1). However, in those with anal CDAC who were well‐known as having poor outcome,^14^ the only significant difference in outcome by behavior is in the RFS analysis evaluated by penetrating or not (Figure S2). A classification system that takes into account not only the concept of CD as a benign disease but also the oncological prognosis of CDAC may be useful in determining future treatment strategies.

Limitations of this study include the following. First, the study was a retrospective study. Second, our data collection spans a long period of time, from 1985 to 2019, treatment policy such as CD classification and the definitions of clinical terms may be ambiguous in some respects depending on the historical background. Third, the indications for surgery and surgical procedures are not standardized due to the number of participating institutions. Fourth, as mentioned above, surgery is not always preceded by the assumption of the presence of cancer. The surgical procedure differs between cases that underwent surgery with a diagnosis of cancer and those that were diagnosed with cancer after surgery, and the difference in clinical course may affect prognosis.

In conclusion, our study highlights the different characteristics of CDAC according to the underlying disease behavior, elucidating the poor prognosis of CDAC patients with penetrating (B3) behavior. As the number of CDAC cases is expected to increase in the future, physicians may contribute to improved prognosis by developing a treatment plan for each case based on the individual's disease behavior.

## AUTHOR CONTRIBUTIONS

Conception and design, acquisition of data, and analysis and interpretation of data: Akira Yamamoto, Yuji Toiyama, Hiroki Ikeuchi, Motoi Uchino, Kitaro Futami, Kinya Okamoto, Takayuki Ogino, Soichiro Ishihara, Yoichi Ajioka, and Kenichi Sugihara; drafting the work critically for important intellectual content: Akira Yamamoto, Yuji Toiyama, Hiroki Ikeuchi, Motoi Uchino, Kitaro Futami, Kinya Okamoto, Takayuki Ogino, Soichiro Ishihara, Yoichi Ajioka, and Kenichi Sugihara; final approval of the version to be published: Akira Yamamoto, Yuji Toiyama, Hiroki Ikeuchi, Motoi Uchino, Kitaro Futami, Kinya Okamoto, Takayuki Ogino, Soichiro Ishihara, Yoichi Ajioka, and Kenichi Sugihara; agreement to be accountable for all aspects of the work: Akira Yamamoto, Yuji Toiyama, Hiroki Ikeuchi, Motoi Uchino, Kitaro Futami, Kinya Okamoto, Takayuki Ogino, Soichiro Ishihara, Yoichi Ajioka, and Kenichi Sugihara.

## FUNDING INFORMATION

This work was supported by the Japanese Society for Cancer of Colon and Rectum.

## CONFLICT OF INTEREST

The authors declare no conflicts of interest for this article.

## ETHICS STATEMENT

Approval of the research protocol: This multicenter retrospective study was approved with the permission of The University of Tokyo Ethics Review Board (2019220NI‐(2)).

Informed Consent: N/A.

Registry and the Registration No. of the study/trial: N/A.

Animal Studies: N/A.

## Supporting information


Data S1
