# Supplementary material for: Oncological outcomes of Crohn's disease‐associated cancers focusing on disease behavior
Source: Ann Gastroenterol Surg. 2023 Jan 18;7(4):615–25. doi: 10.1002/ags3.12653 (PMC10319610; doi:10.1002/ags3.12653)
Supplement: Supplementary file 1 — Data S1 [file AGS3-7-615-s001.docx]

**Supplemental Content**

**Supplementary Figure1.** Oncological outcome of Crohn’s disease-associated colorectal cancer evaluated by disease behavior.

**Supplementary Figure2.** Oncological outcome of Crohn’s disease-associated anal cancer evaluated by disease behavior.

**Supplementary Table1.** Patients' characteristics only among Crohn’s disease-associated colorectal cancer patients.

**Supplementary Table2.** Patients' characteristics only among Crohn’s disease-associated anal cancer patients.


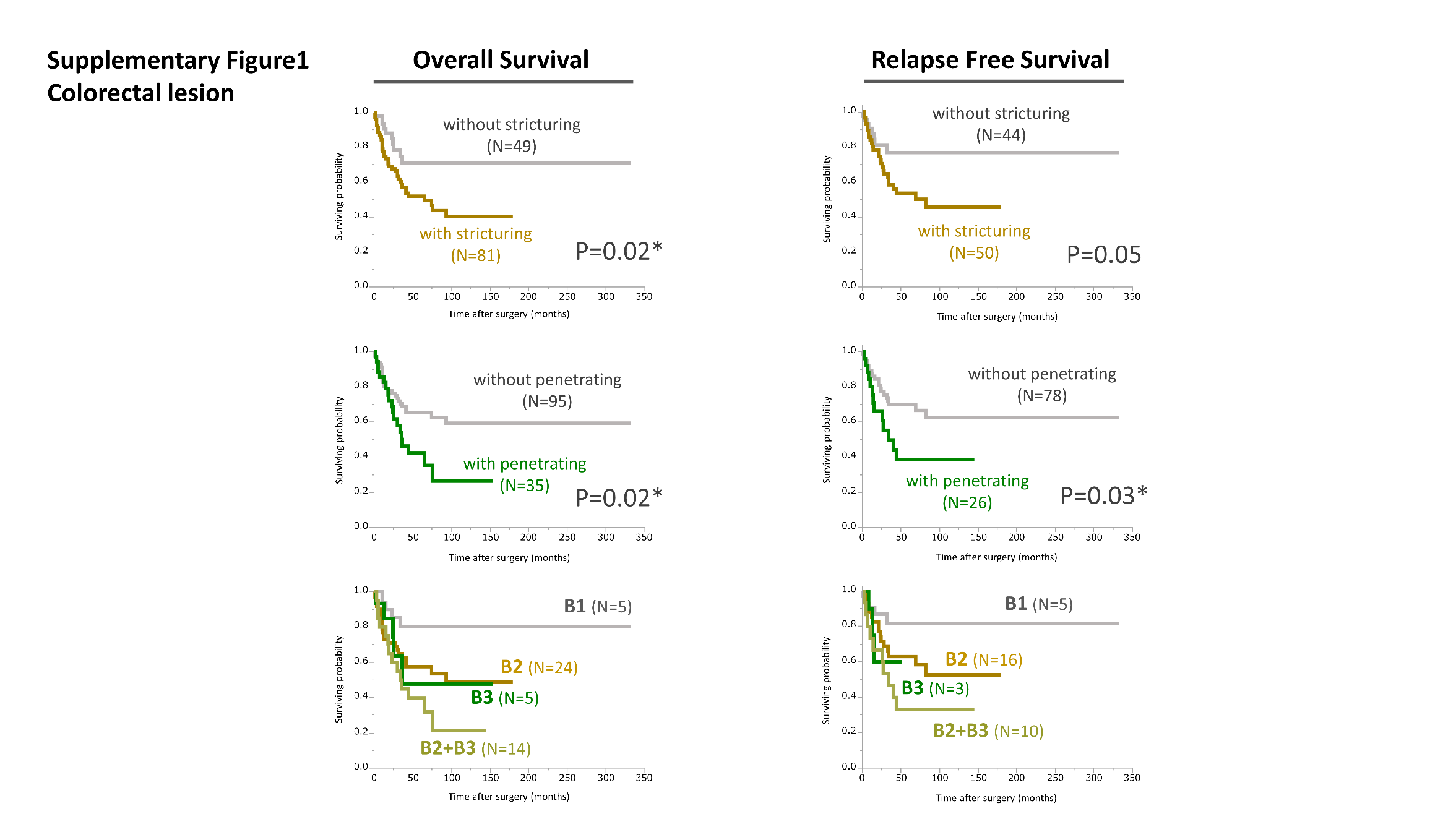


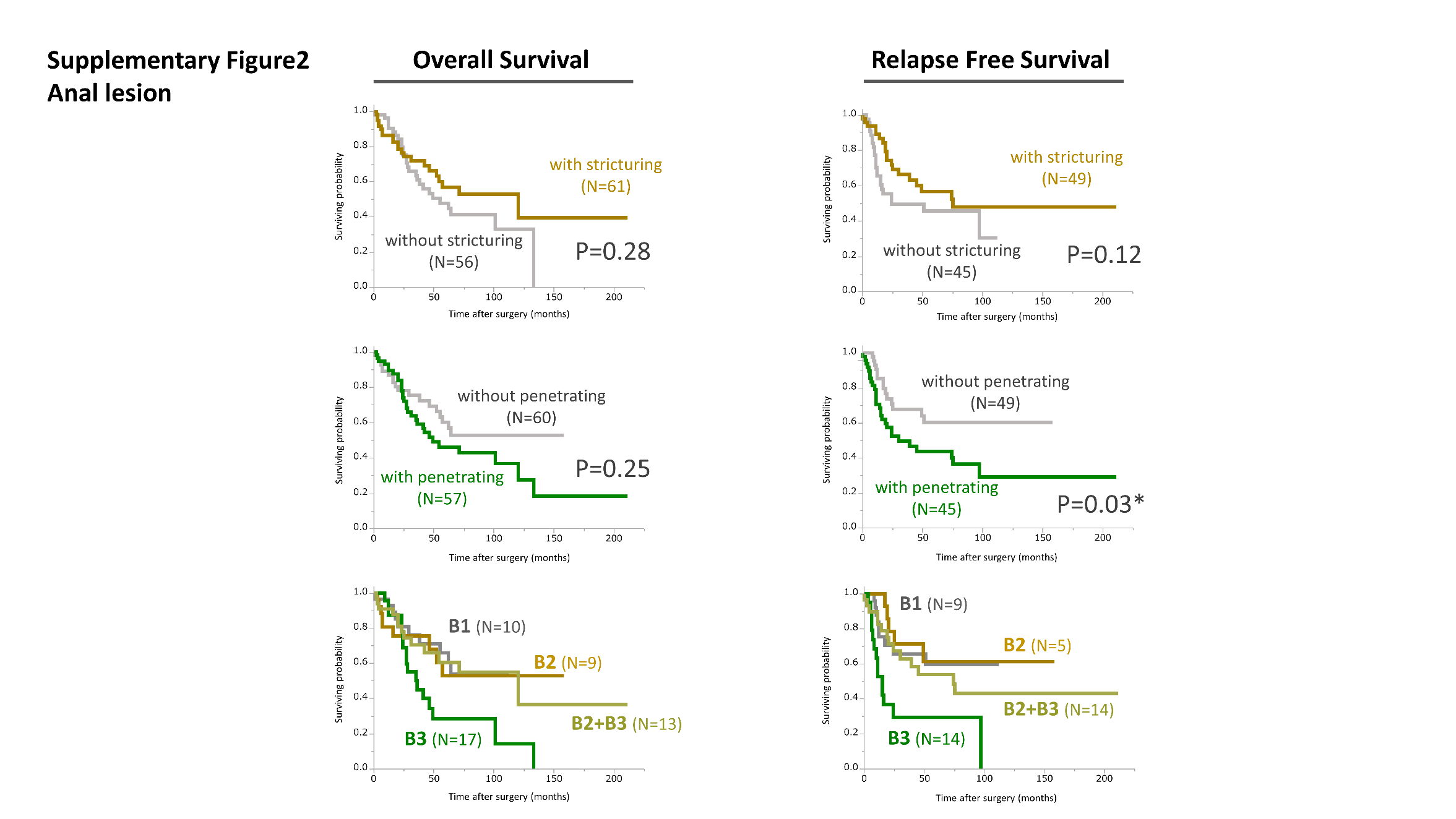


| **Supplementary Table 1. Patients' characteristics (only colorectal lesion)** | | | |  |  |  |  |  |
| --- | --- | --- | --- | --- | --- | --- | --- | --- |
|  |  |  |  |  |  |  |  |  |
|  |  | stricturing | | P value |  | penetrating | | P value |
|  |  | absence | presence |  |  | absence | presence |  |
| Sex | male | 46 (73.2) | 64 (71.1) | 0.8 |  | 77 (70.0) | 33 (76.7) | 0.4 |
|  | female | 17 (27.0) | 26 (28.9) |  |  | 33 (30.0) | 10 (23.3) |  |
| Age at onset of CD | <median | 27 (44.3) | 36 (40.5) | 0.64 |  | 42 (38.9) | 21 (50.0) | 0.22 |
|  | ≥median | 34 (55.7) | 53 (59.5) |  |  | 66 (61.1) | 21 (50.0) |  |
| Disease duration | <median | 35 (40.7) | 41 (53.3) | 0.48 |  | 59 (60.8) | 17 (43.6) | 0.07 |
|  | ≥median | 24 (40.7) | 36 (46.7) |  |  | 38 (39.2) | 22 (56.4) |  |
| Age at diagnosis of Cancer | <median | 29 (47.5) | 41 (48.8) | 0.88 |  | 50 (48.1) | 20 (48.8) | 0.94 |
|  | ≥median | 32 (52.5) | 43 (51.2) |  |  | 54 (51.9) | 21 (51.2) |  |
| Smoking history | no | 35 (70.0) | 49 (72.1) | 0.81 |  | 58 (66.7) | 26 (83.9) | 0.06 |
|  | yes | 15 (30.0) | 19 (27.9) |  |  | 29 (33.3) | 5 (16.1) |  |
| Histology | well to mod. | 32 (62.8) | 34 (43.6) | **0.03*** |  | 53 (57.0) | 13 (36.1) | **0.03*** |
|  | others | 19 (37.2) | 44 (56.4) |  |  | 40 (43.0) | 23 (63.9) |  |
| T factor | T1/2 | 25 (46.3) | 16 (21.3) | **0.002*** |  | 32 (33.3) | 9 (27.3) | 0.52 |
|  | T3/4 | 29 (53.7) | 59 (78.7) |  |  | 64 (66.7) | 24 (72.7) |  |
| Lymphatic invasion | absence | 33 (61.1) | 37 (52.1) | 0.31 |  | 52 (55.3) | 18 (58.1) | 0.79 |
|  | presence | 21 (38.9) | 34 (47.9) |  |  | 42 (44.7) | 13 (41.9) |  |
| Venous invasion | absence | 37 (68.5) | 39 (54.9) | 0.12 |  | 55 (58.5) | 21 (67.7) | 0.36 |
|  | presence | 17 (31.5) | 32 (45.1) |  |  | 39 (41.5) | 10 (32.3) |  |
| Node involvement | absence | 35 (71.4) | 52 (69.3) | 0.8 |  | 62 (66.0) | 25 (83.3) | 0.06 |
|  | presence | 14 (28.6) | 23 (30.7) |  |  | 32 (34.0) | 5 (16.7) |  |
| CD: Crohn's disease. |  |  |  |  |  |  |  |  |
|  | | | |  |  |  |  |  |
| **Supplementary Table 2. Patients' characteristics (only anal lesion)** | | | |  |  |  |  |  |
|  |  |  |  |  |  |  |  |  |
|  |  | stricturing | | P value |  | penetrating | | P value |
|  |  | absence | presence |  |  | absence | presence |  |
| Sex | male | 34 (56.7) | 40 (63.5) | 0.44 |  | 35 (57.4) | 39 (62.9) | 0.53 |
|  | female | 26 (43.3) | 23 (36.5) |  |  | 26 (42.6) | 23 (37.1) |  |
| Age at onset of CD | <median | 31 (51.7) | 45 (71.4) | **0.02*** |  | 36 (59.0) | 40 (64.5) | 0.53 |
|  | ≥median | 29 (48.3) | 18 (28.6) |  |  | 25 (41.0) | 22 (35.5) |  |
| Disease duration | <median | 22 (39.3) | 22 (37.3) | 0.66 |  | 21 (36.2) | 23 (40.4) | 0.65 |
|  | ≥median | 34 (60.7) | 37 (62.7) |  |  | 37 (63.8) | 34 (59.6) |  |
| Age at diagnosis of Cancer | <median | 30 (50.9) | 32 (51.6) | 0.93 |  | 29 (48.3) | 33 (54.1) | 0.53 |
|  | ≥median | 29 (49.2) | 30 (48.4) |  |  | 31 (51.7) | 28 (45.9) |  |
| Smoking history | no | 35 (71.4) | 45 (81.8) | 0.21 |  | 35 (72.9) | 45 (80.4) | 0.37 |
|  | yes | 14 (28.6) | 10 (18.2) |  |  | 13 (27.1) | 11 (19.6) |  |
| Histology | well to mod. | 20 (38.5) | 16 (28.6) | 0.28 |  | 16 (31.4) | 20 (35.1) | 0.68 |
|  | others | 32 (61.5) | 40 (71.4) |  |  | 35 (68.6) | 37 (64.9) |  |
| T factor | T1/2 | 18 (33.3) | 11 (20.8) | 0.14 |  | 18 (36.0) | 11 (19.3) | 0.05 |
|  | T3/4 | 36 (66.7) | 42 (79.2) |  |  | 32 (64.0) | 46 (80.7) |  |
| Lymphatic invasion | absence | 32 (61.5) | 30 (61.2) | 0.97 |  | 30 (63.8) | 32 (59.3) | 0.64 |
|  | presence | 20 (38.5) | 19 (38.8) |  |  | 17 (36.2) | 22 (40.7) |  |
| Venous invasion | absence | 37 (71.2) | 28 (57.1) | 0.14 |  | 30 (63.8) | 35 (64.8) | 0.92 |
|  | presence | 15 (28.6) | 21 (42.9) |  |  | 17 (36.2) | 19 (35.2) |  |
| Node involvement | absence | 37 (68.5) | 33 (66.0) | 0.78 |  | 33 (66.0) | 37 (68.5) | 0.78 |
|  | presence | 17 (31.5) | 17 (34.0) |  |  | 17 (34.0) | 17 (31.5) |  |
| CD: Crohn's disease. |  |  |  |  |  |  |  |  |
